# Bioactive Polyphenols and Neuromodulation: Molecular Mechanisms in Neurodegeneration

**DOI:** 10.3390/ijms21072564

**Published:** 2020-04-07

**Authors:** Francesco Di Meo, Anna Valentino, Orsolina Petillo, Gianfranco Peluso, Stefania Filosa, Stefania Crispi

**Affiliations:** 1 Institute of Biosciences and BioResources-UOS Naples CNR, Via P. Castellino, 111, 80131 Naples, Italy; francesco.dimeo.90@gmail.com; 2 Department of Biology, University of Naples Federico II, Complesso Universitario Monte Sant’Angelo Via Cinthia, 80126 Naples, Italy; 3 Elleva Pharma s.r.l., Via P. Castellino 111, 80131 Naples, Italy; anna.valentino@ellevapharma.com; 4 Institute on Terrestrial Ecosystems (IRET) CNR, Via P. Castellino 111, 80131 Naples, Italy; orsolina.petillo@cnr.it (O.P.); gianfranco.peluso@cnr.it (G.P.); 5 IRCCS Neuromed, Localitá Camerelle, 86077 Pozzilli (IS), Italy; 6 Stazione Zoologica Anton Dohrn, Villa Comunale, 80121 Napoli, Italy

**Keywords:** polyphenols, neuroprotection, apoptosis prevention, oxidative stress, gut microbiota

## Abstract

The interest in dietary polyphenols in recent years has greatly increased due to their antioxidant bioactivity with preventive properties against chronic diseases. Polyphenols, by modulating different cellular functions, play an important role in neuroprotection and are able to neutralize the effects of oxidative stress, inflammation, and apoptosis. Interestingly, all these mechanisms are involved in neurodegeneration. Although polyphenols display differences in their effectiveness due to interindividual variability, recent studies indicated that bioactive polyphenols in food and beverages promote health and prevent age-related cognitive decline. Polyphenols have a poor bioavailability and their digestion by gut microbiota produces active metabolites. In fact, dietary bioactive polyphenols need to be modified by microbiota present in the intestine before being absorbed, and to exert health preventive effects by interacting with cellular signalling pathways. This literature review includes an evaluation of the literature in English up to December 2019 in PubMed and Web of Science databases. A total of 307 studies, consisting of research reports, review articles and articles were examined and 146 were included. The review highlights the role of bioactive polyphenols in neurodegeneration, with a particular emphasis on the cellular and molecular mechanisms that are modulated by polyphenols involved in protection from oxidative stress and apoptosis prevention.

## 1. Introduction

An improvement in socio-economic conditions, especially in developed countries, results in an increase in elderly people. This determines a corresponding enlargement of the pathologies linked to brain aging, such as cognitive and neurodegenerative diseases. The potential mechanisms underpinning brain neurodegeneration have not yet been completely elucidated; nevertheless, oxidative stress and inflammation are considered the main effectors of brain decline.

Nowadays, there is a growing interest in dietary polyphenol nutrients since different epidemiological studies have suggested that diets rich in plant-derived phytochemicals and, in particular, polyphenols, are beneficial to human health [1]

Among phytochemicals, polyphenols are the major group of compounds produced by plants as secondary metabolites that protect plants against reactive oxygen species (ROS), ultraviolet radiation (UV), pathogens, parasites and plant predators. They act as natural antioxidants thanks to their metal-chelating and free radical scavenger properties. Bioactive polyphenols have been reported to prevent the age-related cognitive decline typical of neurodegenerative diseases. Dietary bioactive polyphenols can also modulate cognitive deficits and synaptic plasticity and promote neurogenesis [2,3].

Neurodegenerative diseases are characterized by the progressive loss of function of a specific population of neurons and neural stem cells that results in sensory and motor deficits and cognitive impairment. Different signaling pathways are involved in neurodegeneration, including oxidative stress, inflammation and apoptosis. Bioactive polyphenols are able to counteract these processes by directly scavenging free radical species inhibiting ‘pro-oxidant’ enzymes, activating anti-oxidant enzymes [4] or intervening in apoptotic pathways [5].

Polyphenols act by preventing the DNA damage triggered by hydrogen peroxide (H_2_O_2_) and by transition metals such as copper and iron. Iron-mediated oxidative DNA damage by hydroxyl radical (^•^OH) is the primary cause of cell death under oxidative stress conditions for both prokaryotes and eukaryotes [6]. Polyphenols can also directly scavenge ROS or inhibit the expression of molecules sensitive to oxidative stress such as nuclear factor-kB (Nf-κB) and activator protein-1 (AP-1) [7].

The molecular mechanism of neuroprotection involves the regulation of the mitochondrial apoptosis cascade, which is finely tuned by the imbalance by between B-cell lymphoma-2 (Bcl-2) and Bcl-2-associated X protein (Bax). Phytochemical neuroprotection can be achieved by introducing anti-apoptotic Bcl-2, thus preventing apoptosis [8].

Bioactive polyphenols can exert neuromodulatory effects, activating different intracellular signaling pathways that are crucial for neuroprotection. The PI3K/Akt (Phosphoinositide 3-kinases/Akt) pathway prevents apoptosis by upregulating the expression of Bcl-2; the PKC-ERK1/2 (Protein kinase C/ Extracellular signal-regulated protein kinases 1 and 2) pathway decreases Bcl-2 (B-cell lymphoma 2), Bcl-w (Bcl-2-Like Protein 2) and Bcl-xL (B-cell lymphoma-extra large), which are other anti-apoptotic proteins; Akt-ERK1/2 inhibits the pro-apoptotic activity of Bad (BCL2 associated agonist of cell death) and Bim (Bcl-2-interacting mediator) and activates caspases 9 and 3 [9,10].

The neuroprotective effects of polyphenols can be carried out through synthesis of neurotrophic factors, such as brain-derived neurotrophic factor (BDNF), nerve growth factor (NGF) or glial cell line-derived neurotrophic factor (GDNF). They can exert neurotrophic activity by binding the cognate receptors and activating the downstream neuroprotective pathways [11].

The beneficial health effects of bioactive phenolic compounds mainly depend on their bioavailability and absorption rate. These characteristics can be modulated through interaction with other dietary nutrients and through the action of enzymes present in the small intestine and in the liver. Polyphenols are usually recognized as xenobiotics by the human body. After ingestion, their absorption through the gut barrier can be increased following specific biotransformation and conjugation such as methylation, sulfation and glucuronidation [12].

Polyphenols are effective in neuroprotection in a different way—directly, by affecting brain functions and indirectly, by modulating gut microbiota composition and the metabolites produced. Both actions determine the production of neurotransmitters and neuropeptides that are able to influence the brain functions.

In this review, we discuss the different cellular and molecular mechanisms by which dietary bioactive polyphenols exert neuroprotective functions. The review evaluates the main publications in the field, providing a comprehensive analysis of the efficacy of bioactive polyphenols.

## 2. Methodology

We performed a literature review, searching within the PubMed and Web of Science database. As keywords, we used the terms: oxidative stress, antioxidant, polyphenols, neurodegeneration, neuroprotection, Alzheimer’s disease, Parkinson’s disease, and Huntington’s disease. Research reports, review articles and articles in English published up to December 2019 were selected and evaluated. In addition, we examined the citations therein and included them when appropriate. In total, we examined 307 references and included 146 of them in the present review.

## 3. Oxidative Stress and Polyphenols 

Oxidative stress occurs when ROS accumulate in the cells for excessive production or insufficient neutralization. ROS are a contradiction for the cells. They are produced in normal metabolism as part of several physiologic processes and, when present in physiologic concentrations, they control redox homeostasis in the cell. However, an imbalance between their production and the ability of the cell to enforce antioxidant defense mechanisms may affect cellular structure and functional integrity, resulting in cell damage and leading to necrotic or apoptotic cell death [13,14,15]. 

ROS cause severe molecular damage in the major cell components such as protein oxidation, lipid oxidation, DNA oxidation, and glycoxidation. They can be divided into free radicals and nonradicals. Free radicals are the molecular species containing at least one unpaired electron in the shell around the atomic nucleus.

Among free radicals, H_2_O_2_ is one of the most important ROS with a physiological significance. H_2_O_2_ is formed in the cells in a reaction catalyzed by the superoxide dismutase enzyme (SOD) and also, at relatively low concentrations, it can penetrate the biological membranes and cause severe damage to cellular macromolecules. ROS increase with exposure to the environment. To counterbalance the effect of oxidants, cells have evolved an intricate network of defense mechanisms. Indeed, depending on the intensity and duration of oxidative stress, the cell response can be different, ranging from cell proliferation to cell cycle arrest or cell death by apoptosis or necrosis [16]. 

The adverse effects of ROS can be inactivated by the action of antioxidants [17]. They are defined as compounds able to inhibit the oxidation of any molecule, even when present at very low concentration [18]. By inhibiting or quenching free radical reactions, antioxidants delay or block cellular damage. 

It is widely accepted that neurodegenerative development is associated with oxidative stress that determines severe injury in the cell. In fact, oxidative stress can result in deep biological damage. An imbalance in redox regulation determines the overproduction of ROS with the induction of progressive cellular damage, which is fundamental in neurodegenerative diseases. The production of excessive ROS in neuronal tissue is mainly due to the activity of excitatory amino acids and neurotransmitters [19]. Thus, neuronal cells need antioxidants to scavenge and prevent the formation of ROS.

The body protects itself from ROS by using enzymatic antioxidant mechanisms [20]. The antioxidant enzymes reduce the levels of lipid hydroperoxide and H_2_O_2_; thus, they are important in preventing lipid peroxidation and maintaining the structure and function of cell membranes.

The nonenzymatic antioxidants are the natural antioxidants present in plants. Among them, polyphenols are the most powerful antioxidants able to balance the cellular ROS by activating antioxidant signaling pathways [21,22]. Different studies indicate that dietary antioxidants consumed daily exert a protective role [23].

Plants produce nonenzymatic antioxidants such as vitamins that act to interrupt free radical chain reactions. Vitamin C is present in all plant cells and it has intracellular and extracellular antioxidant capacity. Vitamin E is present exclusively in plastids. It represents the principal defense against oxidative membrane damage, being concentrated in the hydrophobic bilayer of the cell membrane. Vitamin E avoids lipid peroxidation, thus protecting polyunsaturated fatty acids (PUFA) from reactive oxygen damage [24].

**Figure 1:**
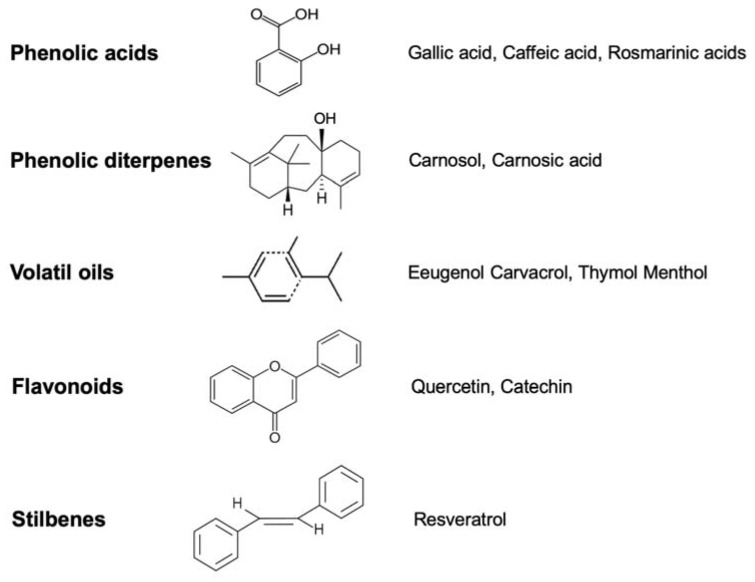
Typical representatives of antioxidant polyphenol classes with their basic chemical structure.

Antioxidant plant polyphenols belong to different groups: phenolic acids, phenolic diterpenes, volatile oils, flavonoids and stilbene (Figure 1) [25]. All act as ROS scavengers, or induce antioxidant enzymes [26].

Phenolic acids exert their antioxidant action, trapping free radicals [27]. The antioxidant mechanism of phenolic diterpenes is related to the scavenging activities of lipid free radicals and to the inhibition of low-density lipoprotein oxidation [28]. Volatile oils, as well as phenolic compounds, act as antioxidants by reacting with peroxyl radicals [29]. Flavonoids are the most abundant class of plant polyphenols, due to their metal-chelating and free radical scavenger properties [30]. On the contrary, stilbenes scavenge hydroxyl radicals.

Other non-flavonoid polyphenols present in foods have antioxidant properties. Among them, curcumin, from turmeric Curcuma longa, prevents lipid peroxidation by scavenging H_2_O_2_ and hydroxyl radicals. Curcumin can also act as an antioxidant, reducing ferric ion (Fe^3+^) and chelating ferrous ion (Fe^2+^) [31].

## 4. Molecular Pathways Involved in Neuroprotection of Polyphenols

Although cells are equipped with a high variety of antioxidants, some tissues and organs are much more vulnerable than others to oxidative stress, probably because of their elevated consumption of oxygen and the consequent generation of large amounts of ROS. Several observations suggest that the brain is particularly vulnerable to oxidative stress [32] and oxidative stress has been implicated in the pathogenesis of many clinical conditions and in the process of aging. 

Oxidative stress has been found to be increased in several human age-related degenerative diseases, including genetically-linked neurodegenerative diseases, like Alzheimer’s disease (AD), Parkinson’s disease (PD), and Huntington’s disease (HD) [33]. However, whether oxidative stress is a primary cause or a downstream consequence of these neuropathological conditions or, more in general, of the aging process is still an open question [34].

Neurons are particularly sensitive to oxidative stress because, being postmitotic cells, they will not be replaced. Moreover, the ability of cells to respond to oxidative protein damage also seems to decline with age [35]. Since the antioxidant systems are overwhelmed in pathological conditions, the use of natural molecules like polyphenols can be viewed as a novel antioxidant therapeutic strategy to reduce neuronal ROS and ameliorate the neurodegenerative process.

Polyphenols, due to their ability to modulate multiple cellular processes including redox balance, have invaluable potential as antioxidant agents. 

Polyphenols such as resveratrol from grapes and wine, curcumin from tumeric, and epigallocatechin-3-gallate from green tea (EGCG), are able to protect against neurodegenerative diseases by activating the protein kinases signaling molecular pathways such as Keap1/Nrf-2/ARE, the major protective pathway against endogenous and exogenous ROS [36,37]. The interaction between Keap1 and bioactive molecules leads to the disruption of the Keap1/Nrf2 complex, allowing Nrf2 to translocate to the nucleus where it binds adenylate and uridylate (AU)-rich elements (AREs) and triggers the expression of antioxidant proteins such as heme oxygenase-1 [38].

Polyphenols also stimulate neurotrophic receptor factors with a pivotal role in the maintenance of neuronal health. Examples are BDNF, which is involved in learning and memory [39], NGF, which is crucial for the survival of brain neurons [40], and GDNF, which regulates cell survival and synaptic plasticity [41]. Several studies reported that different flavonoids, including resveratrol, are able to enhance the expression of these factors. Quercetin and genistein were shown to stimulate NGF-induced neurite outgrowth [42,43]. Resveratrol is able to induce the release of BDNF and GDNF, thus protecting cells from neurotoxicity [44,45].

The neuroprotective role of polyphenols is carried out by activating the pathways that, in cells, regulate transcription, translation, proliferation, growth and survival, such as PI3K/Akt, PKC-ERK1/2, Akt-ERK1/2 and MAPK (Mitogen-activated protein kinase). Their binding to Trk receptors (tyrosine receptor kinases) activates the protein kinase cascades and, finally, cAMP (3’,5’-cyclic adenosine monophosphate)response element-binding protein (CREB), increasing the expression of Bcl-2, Bcl-xL and of neurotrophic factors [46]. In addition, this binding promotes receptor dimerization and autophosphorylation by activating the downstream signaling cascades and promoting the survival of motor neurons, hippocampal neuronal cells, and spinal ganglion neurons [47].

A flavone derivative has been reported to act as a TrkB agonist and to activate the downstream signaling pathway [48]. More recently, resveratrol was shown to exert neuroprotection through its interaction with Trk receptors [49].

In the brain, the anti-inflammatory action of polyphenols leads to neuroprotective effects. In fact, polyphenols inhibit the release of cytokines from activated glia and they also downregulate pro-inflammatory transcription factors such as NF-κB [50]. For example, quercetin determines a strong neuroprotective effect, repressing NF-κB [51]; catechin, after stress injury, increases cell survival by downregulating the NF-κB and MAP kinase pathways [52]. 

**Figure 2:**
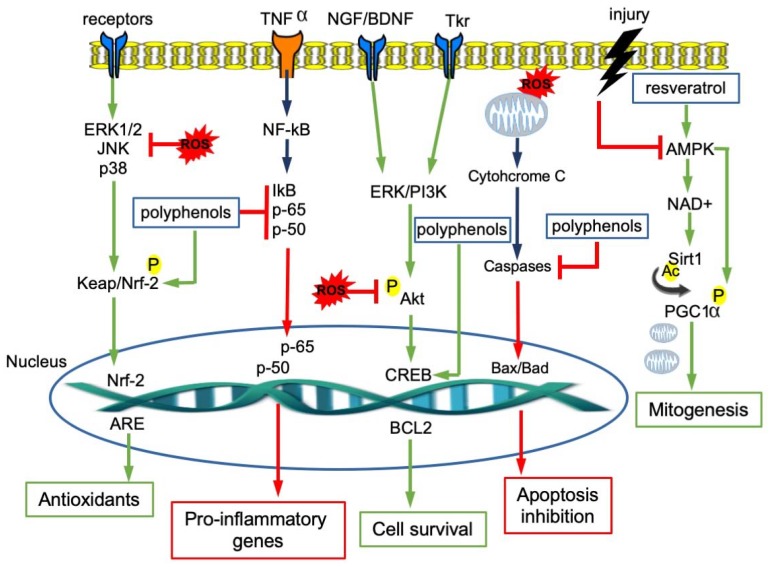
Intracellular signaling pathways involved in neuroprotection and modulated by polyphenols.

The molecular mechanisms modulated by polyphenols are summarized in Figure 2.

## 5. Effect of Polyphenols on Neurodegenerative Disorders

Despite the variability in the clinical picture of neurodegenerative disorders, these diseases share common molecular traits. Inflammation and oxidative stress are responsible for the disruption of the functions of the neurovascular units in AD, PD, HD and dementia. ROS are able to interact with different neuronal signaling pathways, such as protein kinase and lipid kinase signaling cascades [53,54] (Table 1).

### 5.1. Alzheimer’s Disease 

AD is the most frequent form of dementia in the elderly population [63], with progressive neurodegeneration. It is characterized by the deposition of Amyloid β (Aβ) peptides as Aβ plaques and intracellular neurofibrillary tangles [64,65] that ultimately lead to a gradual deterioration in brain structure and to the loss of intellectual function [66].

The presence of amyloid beta (Aβ) peptides in Alzheimer’s disease confers oxidative insult on neurons and glial cells, leading to a change in synaptic plasticity [67]. The neuronal cytotoxicity in AD seems to be imputable to N-methyl- D-aspartic acid (NMDA) receptor activation coupled with ROS production [68]. This mechanism, through the PKC/MAPK pathways, leads to the release of arachidonic acid, involved in AD neuron apoptosis. Aβ peptides and ROS promote neurotoxicity in AD, inactivating PI3k/Akt pathways. The Akt inactivation regulates various pro-apoptotic mediators [56]. Several studies indicated that a diet rich in polyphenols inhibits the above-mentioned pathways [69]. Curcumin displays anti-amyloidogenic properties, preventing the neurodegenerative process in AD through the inhibition of MAPK and PI-3K pathways [70]. In addition, curcumin is able to block the BDNF decrease in rats inoculated with Aβ peptide, modulating the Akt/GSK-3β signaling pathway, thus determining a cognitive improvement [71]. Recently, it has been reported in an AD mouse model that curcumin reduced Aβ production through the inhibition of β-secretase (BACE-1), the enzyme that is responsible for the proteolytic processing of the amyloid precursor protein [72].

The antioxidant activity of resveratrol protects the memory decline in AD. In cells, it has been reported that resveratrol suppresses Aβ-induced ROS generation and apoptosis [73]. Another study reported that resveratrol exerts a neuroprotective role through its modulation of the PI3K/Akt signaling pathway [74]. SIRT1 (Sirtuin 1) in brain has been shown to be protective against neurodegeneration by deacetylating several transcription factors involved in neuronal protection and stress resistance [75]. Dietary resveratrol protects against Aβ formation and oxidative stress by modulating SIRT1 expression [76,77]. In particular, this ability is linked to the deacetylation of PGC-1α, to the presence of active proliferator-activated receptor-γ (PPAR-γ), and to the protection against mitochondrial damage by Bcl-2 upregulation [55]. EGCG was described as active towards AD in animal studies, being able to significantly reduce the cognitive decline and Aβ peptides, and to upregulate proteins related to synaptic plasticity [78]. EGCG prevents neuronal apoptosis from neurotoxic processes inhibiting BACE-1 [79]. In addition, EGCG significantly improves neuronal survival and hippocampal neurogenesis by activating the PI3K/Akt signaling pathway and inhibiting the MAPK pathway [80]. In vitro and in vivo studies reported the neuroprotective role of quercetin against Aβ toxicity, showing that this polyphenol determined cell viability increase with a reduction in neuronal oxidative stress [81]. Quercetin seems to inhibit the Aβ plaque aggregation and the formation of neurofibrillary tangles probably increasing the levels of apolipoprotein E, that has a key role in the clearance of Aβ [82]. A study performed in a triple transgenic mouse model of AD showed that quercetin, after three months treatment, was able to reduce the amount of the β-amyloid fibers with improvement of cognitive performances [83]. More recently, it was shown that a quercetin-enriched diet affected the latency in APP/PS1 mice only when administered at early stage. The effect was due to the inhibition of amyloidogenic processing through the reduction in the BACE-1 enzyme [84]. Finally, quercetin treatments in Drosophila models of AD indicated that this flavonoid could restore Aβ-induced perturbation by acting on cell cycle signaling pathways and DNA replication [85].

### 5.2. Parkinson’s Disease

PD is a chronic and long-term degenerative disorder, characterized by the loss of dopaminergic neurons in the substantia nigra, which determines clinical motor deficits such as rigidity, bradykinesia and tremors [57].

Oxidative stress remains the strongest leading theory to explain the progressive loss of dopaminergic neurons in substantia nigra in PD patients [58]. Microglia produce high levels of ROS through NADPH oxidase, which induces the PKC delta and ERK1/2 pathways’ activation of genes involved in apoptosis [59]. The neuroprotective effect of polyphenols, has been linked to their free radical scavenging and anti-inflammatory properties, both in cellular and animal models [60,61]. They are able to reduce neurotoxicity by interacting with protein aggregates such as α-synuclein [62].

Different studies using in vitro and in vivo models of toxin- induced PD indicated that curcumin reduces oxidative stress with a reduction in apoptosis through the Akt/Nrf2 signaling pathway, since an increase in antioxidant enzyme activity via Nrf2 transcription was detected [86,87,88]. Several experiments also reported that curcumin in PD is able to counteract the decrease in the enzyme tyrosine hydroxylase—the rate-limiting enzyme involved in dopamine synthesis—which has been suggested to be causative in the onset of PD [89]. Curcumin exerts neuroprotective effects in PD by inhibiting oxidative stress through the decrease in ROS, TNF-α and IL-6 and the concomitant increase in Glutathione (GSH) levels [90]. On the other hand, in PD, curcumin not only acts as an antioxidant, but also as anti-inflammatory, reducing the production of TNF-α and Interleukin-6 (IL-6). The beneficial effect of curcumin in the pathophysiology of PD can be also linked to the ability to decrease toll-like receptor 4 (TLR4) and its downstream effectors (NF-κB, IRF3 (Interferon Regulatory Factor 3) and MyD88 (Myeloid differentiation primary response 88) [91].

Resveratrol seems to be a direct modulator in PD-affected pathways. Numerous in vitro studies have demonstrated that this molecule is able to prevent the rotenone-induced autophagic dysfunction by promoting the degradation of α-synuclein [92]. Other studies showed that resveratrol has a protective effect acting on the AKT/GSK-3β signaling pathway [93] or by inhibiting apoptosis through the upregulation of antioxidant enzymes [94]. In addition, resveratrol neurotrophic effects are accomplished through CREB activation in the hippocampus and amygdala neurons, thus reducing oxidative damage induced by neurotoxins [11]. The antioxidant effects of quercetin in PD have not been fully elucidated. Some studies reported that quercetin protected neurons in a rotenone-induced rat model of PD in a dose-dependent manner, upregulating mitochondrial complex-I activity. This molecular mechanism strongly suggests that quercetin has the ability to repair defective mitochondrial electron transport, a hallmark of PD. In addition, quercetin decreases glutathione levels and increases catalase and superoxide dismutase [95]. EGCG in PD exerts neuroprotective effects through AMPK activation, which positively regulates the mitochondrial biogenesis needed for dopaminergic neuronal survival. EGCG in vivo has been reported to preserve the loss of dopaminergic neurons by inhibiting neuronal nitric oxide synthase [96]. EGCG was also shown to prevent striatal dopamine depletion and dopaminergic neuron loss in substantia nigra [97].

### 5.3. Huntington’s Disease and Vascular Dementia

In addition to AD and PD, two other neurodegenerative disorders, Huntington’s disease and dementia, are characterized by a total compromise in cognition. The role of polyphenols in prevention/treatment is not so extensively studied in these two neurodegenerative diseases. 

HD is a dominantly inherited neurodegenerative disorder characterized by progressive striatal and cortical neurodegeneration with associated motor and cognitive defects. The disease-causing mutation is an expansion of a CAG trinucleotide repeat (>36 repeats) encoding a polyglutamine stretch in the N-terminal region of the huntingtin protein, a ubiquitous protein whose function is still unclear [98]. Mutated Huntington is expressed not only in the brain neurons, but also in the enteric neurons [99,100]. HD has also been associated with mitochondrial dysfunction and oxidative stress, as possible disease mechanisms [101,102]. HD cellular models displayed a deregulation in mitochondrial membrane potential and respiration, implicating a decline in mitochondrial function. It has been reported that resveratrol in HD increases the transcription of genes associated to mitochondrial function [103].

To date, only a few studies have analyzed the effects of curcumin in HD. It has been described that curcumin treatment in a rat model of HD reduced mitochondrial damage and exerted antioxidant effects by activating the Nrf2 pathway [104]. A different study using *Drosophila melanogaster* as an HD model showed that curcumin protects against neurodegeneration, suppressing polyglutamine cytotoxicity and cell death [105]. The neuroprotective role of curcumin has been recently reported in an HD transgenic animal model. This study evidenced that curcumin protected the brain from neuropathological and phenotypic complications associated to the disease [106]. It has been reported that resveratrol in HD increases the transcription of genes associated to mitochondrial function [103]. Resveratrol in HD seems to modulate SIRT. Resveratrol in mouse models of HD has been shown to strongly increase transcription of mitochondrial genes and to enhance mitochondrial function. This activity determines an improvement of motor function in HD transgenic mice [103]. Quercetin was able to reduce mitochondrial oxidative stress in HD. This effect leads to an increase in motor skills and coordination, as reported in a drug-induced HD model. Indeed, a reduction in the neuro-inflammatory response and an increased number of astrocytes and decreased microglial proliferation were observed in core lesions [107,108].

Vascular dementia arises from chronic vascular damages in the brain. Cerebral ischemia, increasing ROS production, induces neuronal injury accompanied by a progressive decline in memory and cognitive function [109]. In dementia, curcumin, resveratrol and catechins act as free radical scavengers, as well as natural anti-inflammatory agents, by suppressing the TNF-mediated NF-κB activation. In addition, they act to upregulate endogenous antioxidant enzymes and downregulate enzymes involved in the production of ROS [110]. Curcumin was shown to restore memory deficit in an induced mouse model of memory impairment, thanks to its antioxidant action and to the improvement of cerebral circulation [111]. Resveratrol was neuroprotective against vascular dementia by reducing cell death in the hippocampus and preventing the loss of reference memory [112]. In a rat model of vascular dementia, resveratrol was able to restore the cognitive deficits, to reverse oxidative stress levels and BDNF depletion [113]. In a mouse model of dementia, treatment with quercetin restored cognitive deficit and energy metabolism by directly scavenging superoxide, hydroxyl radicals and by inhibiting various oxidases [114]. A summary of the effects of polyphenols in neurodegenerative diseases is reported in Table 2.

## 6. Bioactivity of Polyphenols and Gut Microbiota Interplay

**Figure 3:**
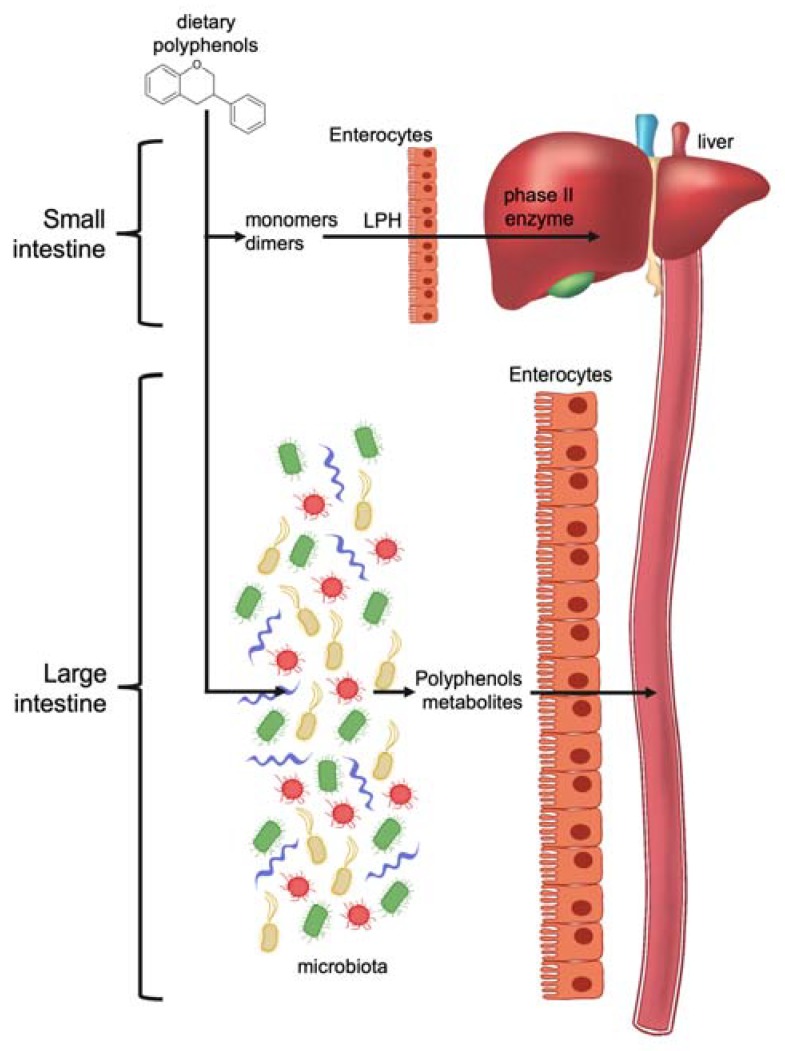
Dietary polyphenol metabolism in small and large intestine. In the small intestine, low molecular weight polyphenols, monomers or dimers, can be absorbed directly or after phase II reaction metabolic conversion. In the large intestine, high molecular weight and conjugated polyphenols are absorbed after transformation processes by enzymes produced by bacteria.

Most of the polyphenols from plants ingested in the diet undergo intestinal transformation before being absorbed by enterocytes and colonocytes. The modifications can be either by enzymes produced by the enterocytes themselves or by the microbiota present in the intestine (Figure 3).

The bioavailability of native polyphenols is very low after recruitment at circulating levels. Polyphenols, to be effective, must be transformed into potentially more bioactive compounds, such as low-molecular weight metabolites [116].

Gut microbiota is essential in transforming numerous compounds that reach the colon, thanks to the capacity of microorganisms to produce a huge and varied range of enzymes.

Enzymatic conjugation processes are needed to reduce the potential toxic effects of polyphenols. These modifications result in the formation of polyphenol metabolites that show new biological activities [117].

The two main factors determining the modification of dietary polyphenols in the gastrointestinal tract are the chemical polyphenols’ structure and the individual variety of gut microbiota composition. In fact, the structural subfamily and its scaffold enable only some transformations, thus reducing the kind (class, species) of final bioactive compounds that are produced. Some modifications of polyphenols can occur through enzymes produced by the majority of bacteria, while others require enzymes produced by specific bacterial species. The presence or absence of these bacteria in the individual’s microbiota will cause different biotransformation of dietary polyphenols. This means that beneficial effects for consumers depend on the dietary polyphenols and on the individual’s microbiota composition.

Polyphenols are molecules with high complexity and for this reason they generally reach the large intestine without modifications. The most complex oligomers are not absorbed in the small intestine, but are processed in the colon by microbiota [118].

EGCG, for example, is transformed in aglycones and gallate, which is further decarboxylated into pyrogallol. In vitro pyrogallol drastically inhibits monocyte migration by reducing levels of inflammatory macrophage differentiation. In addition, pyrogallol increases the phosphorylation of PI3K-AKT and AMPK and reduces caspase levels. In this way, it inhibits monocyte-associated cell death [119]. Pyrogallol is also a GPR35 agonist. GPR35 is an orphan G protein-coupled receptor and is associated to inflammation, cardiovascular diseases, metabolic disorders, Parkinson’s disease and other neuronal disorders. GPR35 agonists, as catechol-O-methyl transferase inhibitors, are commonly used for the treatment of Parkinson’s disease [120]. Pyrogallol can also block aggregate formation in α-synuclein [121]. The presence of aberrant soluble oligomeric conformations of α-synuclein may contribute to PD pathogenesis. Another example is curcumin that is modified in the colon tract by a specific enzyme produced by *E. coli*. This bacterium converts curcumin in its active metabolite, tetrahydrocurcumin, in a two-step reduction reaction. This metabolite shows both greater in vitro and in vivo antioxidant activity than curcumin [122]. Resveratrol bioavailability is greatly increased by gut microbiota metabolism. Resveratrol in plants is present as a glycosidic form of piceid, that is a stilbenoid glucoside. Two different bacteria—*Bifidobacteria infantis* and *Lactobacillus acidophilus*—convert piceid into resveratrol [123]. Then, resveratrol is further metabolized to obtain the active aglycone form by *Slackia equolifaciens* and *Adlercreutzia equolifaciens* [124].

Bacteria in the gut can cleave the ring structure of several flavonoids into short-chain fatty acids (SCFAs) like hydroxyphenylacetic and hydroxyphenylpropionic acids, as well as into acetate and butyrate [125]. Some polyphenols, like quercetin, are not modified in SCFAs; nonetheless, they can enhance the production of SCFAs, especially butyric acid [126].

Dietary polyphenols show neuroprotective potential, but their selective permeability across the blood–brain barrier (BBB) limits their bioavailability, thus limiting their protective efficacy. The BBB is a dynamic interface that regulates molecular interactions between the blood and the neuronal tissue, having an essential role in providing nutrients and other molecules and regulating the access of compounds to the brain. After intestinal absorption, some polyphenol metabolites can reach concentrations in the bloodstream that can exert effects in vivo. However, the effective brain uptake of these polyphenols’ metabolites, and their possible direct neuroprotective potential, is still controversial, given that the exact mechanisms by which they may permeate the BBB are not completely understood. Nerveless, polyphenol microbial metabolites largely showed greater permeability through gut and blood–brain barriers compared to their parent compounds. For example, 5-(hydroxyphenyl)-γ-valerolactone-*O*-sulfate, a secondary microbial metabolite of the flavan-3-ols, is able to reach the brain and cross the BBB in in silico, in vitro and in vivo models [127]. 

Microbial metabolites derived from dietary lignans, a class of polyphenols, like equol and enterolactone, passively cross both the gut and BBB barriers and show protective ability against inflammation in microglia [128]. A recent study demonstrates that microbial polyphenol metabolites could be transported across the BBB endothelium [129]. In vitro experiments showed that some polyphenols’ metabolites cross BBB by transmembrane diffusion and their lipophilicity can determine greater or minor uptake [130]. Nevertheless, it is not clear whether the primary route by which polyphenols’ metabolites cross the BBB happens by simple diffusion or by carrier-mediated transport.

Gut microbiota and polyphenols influence each other. Gut microbiota transforms polyphenols, improving bioavailability and health effects. At the same time, dietary polyphenols regulate microbiota composition, favoring the growth of some bacteria and avoiding the growth of pathogens. Specifically, dietary polyphenols have shown the ability to modulate gut microbiota composition and function, interfering with bacterial quorum sensing, membrane permeability, as well as sensitizing bacteria to xenobiotics.

Specific polyphenols modulate microbiota community composition, modifying the ratio of bacteroides/firmicutes, the most frequent bacteria genera present in the distal gut [131]. For example, resveratrol presented a significant antibacterial activity towards clinically important bacteria, such as *Salmonella enterica, Enterococcus faecalis*, and *Escherichia coli* [132]. The effects of polyphenols on gut microbiota have been shown in human studies. Dietary polyphenols’ mechanism of action is different in Gram-positive and Gram-negative bacteria due to changes in cell membrane structure. Polyphenols bind bacterial cell membranes in a concentration-dependent manner; thus, they modify their membrane and alter their growth. Catechins reacted with the dissolved oxygen in aqueous solution, resulting in the generation of hydrogen peroxide. H_2_O_2_ modifies the permeability of the microbial cell membrane, thus sensitizing the bacteria to the effects of antibiotics [133]. 

Thus, different polyphenols can change the composition of the microbiota (Table 3), and different bacterial populations possess different enzymes that can change the metabolism of dietary polyphenols in different ways. For example, the intake of catechins, which occur in green tea and black tea, considerably boosted the growth and development of members of *E. coli*. As mentioned above, *E. coli* is able to modify curcumin in its active metabolite, tetrahydrocurcumin, which has to be anti-inflammatory and neuroprotective. So, polyphenols can modify the composition of gut microbiota which, in turn, produce secondary metabolites that have neuroprotective effects.

However, gut microbiota can have a direct influence on brain function and alterations of the microbiota composition have been found in some neurodegenerative diseases, including PD and AD [140,141]. In addition, microbiota can exert neuroprotective effects by producing neurotransmitters and neuropeptides [142]. It has been shown that Streptococcus, Escherichia and Enterococcus spp. produce serotonin [143,144] and that Bifidobacterium infantis can modulate central serotonin transmission by increasing plasma tryptophan levels [145]. Meanwhile, different Lactobacillus and Bifidobacterium species are able to produce γ-aminobutyric acid (GABA), the principal inhibitory neurotransmitter in the central nervous system [146]. Therefore, gut microbiota can directly or indirectly contribute to neuroprotection.

## 7. Conclusions

The continuous increase in life expectancy is inversely correlated with quality of life during aging. During life there is, particularly in the brain, an accumulation of damage and a decrease in all of the mechanisms needed for cell repair. These processes can induce cell death.

Aging is one of the leading “risk factors” in the development of neurodegenerative processes. Indeed, neurodegenerative diseases are caused by nervous system dysfunction resulting from neuronal cell failure or cell death.

Neurodegenerative diseases increase with age and are becoming a big challenge for modern societies. Actually, neurodegenerative disorders in developed regions will have a strong increasing impact on medical and socio-economic conditions, since the population is becoming older. Therefore, it is essential to find strategies that help to prevent cognitive decline and improve the life quality of people living with dementia.

For thousands of years, it has been well known that food and health are related to each other. Indeed, even Hippocrates (400 BC), emphasized the importance of nutrition to prevent or cure diseases. In more recent years, different epidemiological studies have confirmed this thought, evidencing a strong link between the consumption of polyphenols and neurocognitive protection and suggesting that a polyphenol-rich diet can be an effective strategy to improve cognitive function in elderly populations.

Polyphenols are bioactive compounds contained in in food and beverages that are able to modulate the metabolic process, thus promoting health and preventing the age-related decline of cognitive, motor and sensory activities. Being antioxidants, polyphenols are able to counteract the oxidative damage accumulated in the cells. Moreover, they modulate different cellular signaling pathways, protecting cells from stress injury. Therefore, understanding the mechanisms by which polyphenols act at a molecular level is crucial in order to use them as dietary supplements to prevent neurodegenerative disorders.

Although polyphenols are abundant in fruits and vegetables and their intake can be increased in the diet, their bioavailability is also influenced by their chemical structure. Unlike in vitro, polyphenols in vivo have limited availability and they need to be metabolized rapidly in order to overcome biological barriers. Thus, gut microbiota has a key role in the production of specific bioactive polyphenols’ metabolites that are responsible for these health effects. Moreover, it is emerging that polyphenols can also modulate the bacterial composition of the gut microbiota, thus influencing the production of specific metabolites that act in modulating brain functions.

There is a growing attention being paid to food diversity, to the consumption of food rich in antioxidants and to developing novel food rich in the different bioactive nutrient groups. To date, the analysis of the impact of these products on neurodegeneration is limited, thus there is a need for an increasing number of studies to better evaluate the dose–effect relationship.

In conclusion, polyphenols and their metabolites are essential compounds with multiple biological activities. Their efficacy as antioxidants and their capacity to modulate pro-survival or anti-apoptotic signaling pathways are essential in preventing and slowing down neurodegenerative disorders. Moreover, considering that they are safe and have a very low toxicity, it is easy to test their efficacy in pre-clinical and clinical studies for the treatment of neurodegenerative diseases. Finally, different polyphenols, or their synthetic derivatives, have been patented as drugs against various human diseases in recent years.

## Figures and Tables

**Table 1:** Signaling pathways activated by polyphenols in neurodegenerative diseases.

Polyphenol	Signaling Pathway	References
Resveratrol	SIRT1/PGC-1	[52,55]
	PI3K/Akt	
Curcumin	AMPK/NF-kB	[56,57,58]
	PI3K/Akt/GSK-3β	
Quercetin	MAPK/AKT/ PI3K	[59]
	ERK/CREB	
Catechins (EGCG)	PKC/MAPK/PI3K/Akt	[60,61,62]
	MEK/ERK1/2	

**Table 2:** Summary of the effects of polyphenol treatment from in vitro and in vivo studies.

Pathology	Type of Study	Polyphenols (dose)	Time	Effect	References
Alzheimer’s disease	mice	grape extract (5–20 μM)	5 months	Inhibition of Aβ oligomerization	[3]
Parkinson’s disease	neuroblastoma cell line	caffeic acid (10–100 μM)	1 hour	Prevention of apoptotic cell death	[9]
Neurodegenerative disorders	neonatal mouse cerebellum cells	curcumin (1–50 μM)	24 hours	Enhancement and repair of neural plasticity	[37]
Alzheimer’s disease	rats	curcumin (50–200 mg/kg)	7 days	Improvement of cognitive deficits	[71,72]
CNS disorders	mice	Resveratrol (20 mg/kg)	7 days	Regulation of pathway involved in CNS disorder and aging	[73]
Alzheimer’s disease	mice	Resveratrol (24 mg/kg)	45 days	Anti-oxidant effect against beta-amyloid plaque formation	[76,77]
Alzheimer’s disease	mice	ECGC (50 mg/kg)	6 months	Reduction in A-β deposition	[78]
Alzheimer’s disease	human brain microvascular endothelial cells	Quercetin (0.3–30 μmol/L)	24 hours	Increase in cell viability and of antioxidant activity	[81]
Parkinson’s disease	primary rat mesencephalic cells	Catechin, quercetin (40 μM)	48 hours	Protective effect on DA neurons under oxidative stress	[61,70]
Parkinson’s disease	rodent model	Curcumin 30 mg/kg)	4 days	Neuroprotective actions (anti-inflammatory and anti-oxidative)	[88,91,115]
Parkinson- like disease	dopaminergic-like cells	Resveratrol (50–200 μM)	12 hours	Neuroprotective effects by inhibiting apoptosis caused by oxidative stress	[92]
Parkinson’s disease	rats	Resveratrol (20 mg/kg)	21 days	Prevention of neuronal death	[94,95]
Parkinson’s disease	rats	Quercetin (25–75 mg/kg)	4 days	Neuroprotective effect observed in neurotoxin-induced Parkinsonism	[95]
Parkinson’s disease	mice	ECGC (20 mg/kg)	5 days	Preventive effects on NOS	[96,97]
Huntington’s disease	mice	ECGC (1 mg/kg)	28 days	Improvement of gene transcription associated to mitochondrial function	[103]
Huntington’s disease	rats	Curcumin (40 mg/kg)	7 days	Amelioration of mitochondrial dysfunctions	[104,105]
Huntington’s disease	mice	Curcumin (20–40 mg/kg)	7 days	Alleviation of debilitating symptoms associated with the disease	[106]
Huntington’s disease	rats	Quercetin (25–50 mg/kg)	4 days	Potential use for inflammatory damages	[107,108]
Memory and cerebral blood flow	mice	Curcumin (10–50 mg/kg)	21 days	Beneficial effects of oxidative stress associated with neurodegenerative disorders	[111]
Dementia	rats	Resveratrol (10–20 mg/kg)	4 days	Neurorestorative effects	[113]
Memory dysfunction	mice	Quercetin (2.5–10 mg/kg)	21 days	Protective toward off dementia and neurodegenerative disorders	[114]

**Table 3:** Summary of the main findings of clinical trial studies related to the effects of polyphenols on gut microbiota by increase (+) or decrease (−) of specific strains.

Polyphenols		Bacteria	References
Catechin and epicatechin	+	*Clostridium coccoides–Eubacterium rectale*	[134]
	+	*E. coli*	
	−	*Clostridium histolyticum*	
Proanthocyanidin	+	*Bifidobacteria*	[135]
Pomegranate extract	+	*Odonbacter*	[136]
	+	*Faecalibacterium*	
	+	*Butyricicoccus*	
	+	*Butyricimonas*	
	+	*Bacteroides*	
	−	*Parvimonas*	
	−	*Metanobrevibacter*	
	−	*Metanosphaera*	
Cocoa flavonols	+	*Bifidobacterium*	[137]
	−	*Lactobacillus*	
	−	*Clostridia*	
Red wine	+	*Enterococcus*	[138]
	+	*Prevotella*	
	+	*Bacteroides*	
	+	*Bifidobacterium*	
	+	*Enterococcus*	
	+	*Bacteroides uniformis*	
	+	*Eggerthella lenta*	
	+	*Blautia coccoides*	
Orange juice	+	*Mogibacteriaceae*	[139]
	+	*Tissierellaceae*	
	+	*Veillonellaceae*	
	+	*Odoribacteraceae*	
	+	*Ruminococcaceae*	
